# Changes in optical properties of aesthetic paediatric restorative materials following exposure to beverages: In-vitro study

**DOI:** 10.1007/s40368-024-00986-4

**Published:** 2025-01-07

**Authors:** A. A. Elkhatib, G. E. Elwardani

**Affiliations:** 1 Pediatric Dentistry and Dental Public Health Department, Faculty of Oral and Dental Medicine, Kafrelsheikh University, Kafrelsheikh, Egypt; 2 Pediatric Dentistry and Dental Public Health Department.Faculty of Dentistry, Alexandria University, Alexandria, Egypt; 3 Alexandria University Main Hospitals, Alexandria, Egypt

**Keywords:** Aesthetic, Colour, Translucency, Primary teeth, Beverages

## Abstract

**Purpose:**

Optical properties of recent aesthetic restorative materials must maintain an acceptable appearance throughout their functional lifetime. This study aimed to assess the changes in translucency and colour of recent resin-based restorative materials after exposure to beverages commonly consumed by children.

**Methods:**

An experimental in-vitro study on 48 discs specimens prepared from; Group I: Filtek Z250 XT (Nanohybrid), Group II: Cention N (Alkasite bulkfill), and Group III: SDR flow Plus (Flowable bulkfill). The prepared disc specimens were randomly assigned into 4 subgroups (*n* = 12) according to the immersion solution: Subgroup A (control): distilled water, B: Coca-Cola, C: chocolate milk, and D: Orange juice. Translucency and colour measurements were done before immersion (baseline) and on day 30 of exposure. Statistical analysis involved Two Way Repeated Measures ANOVA and Kruskal–Wallis test.

**Results:**

The statistical analysis revealed that the restorative material, immersion time, and immersion solution had a significant effect on the change in translucency (*p* < 0.0001*, *p* < 0.0001*, *p* = 0.001*, respectively). Regarding colour changes, different immersion solutions had a statistically significant effect on Filtek Z250 XT and Cention N (*p* < 0.0001*). Distilled water and Coca-Cola were found to have a significant effect on colour change for all restorative materials investigated (*p* < 0.0001*, *p* = 0.003*).

**Conclusion:**

the optical properties of resin-based restorative materials used in paediatric dentistry were notably affected by prolonged exposure to beverages commonly consumed by children.

## Introduction

The increasing demand for better aesthetics among children and their parents had made resin-based materials the first choice for restoring primary teeth. Composite resins have been successfully used for restoring primary teeth for years (Bahbishi et al. 2020). One of the modern advances is the application of nanotechnology to resin composites through combining nanofillers and micro-sized particles. Filtek™ Z250 XT is a nanohybrid composite with broad particle size distribution and high filler loading, thus improving both mechanical and optical properties (Jandt and Watts 2020).

Another recent development of resin-based restorative material is the introduction of the alkasite material (Cention N). As the name implies, this material contains alkaline fillers that is capable of releasing fluoride, calcium, and hydroxyl ions resulting in a potent anticariogenic activity (Karakaş and Küden 2023). In which, hydroxyl ions neutralize the acidic attack produced by oral flora, thus preventing demineralization. Whist the release of fluoride and calcium act as a reservoir for remineralization (Alla et al. 2023).

The latest enhancement to composite resins is the arising of the bulk-filling technique (Bahbishi et al. 2020). Bulk-fill resin achieves effective polymerization depth of cure up to 4 mm, reducing the working time required for incremental placement of conventional composites. This feature is of great advantage in non-cooperative paediatric patients (Arregui et al. 2016).

In aesthetic dentistry, restorative materials should not only resemble the appearance of a natural tooth initially but must also maintain an acceptable appearance throughout the functional lifetime of the restoration (Elwardani et al. 2019). Favourable aesthetic material should closely match the natural teeth optical properties, namely translucency and colour (Alla et al. 2023). However, alterations to the optical properties over time is a common challenge facing all restorative materials. Such variation is a result of the dynamic exposure to changes in temperature and acidity, in addition to the continuous intake of coloured food and beverage (Paolone et al. 2022). A recent study showed that carbonated soft drink, as well as sweetened milk and juices are frequently consumed among Egyptian children (Mahmoud et al. 2022).

Thus, the aim of this invitro study was to investigate changes in translucency and colour of recent resin-based restorative materials after exposure to beverages commonly consumed by children. The secondary aim was to compare the effect of different beverages with time on the optical properties of resin based restorative materials. The null hypotheses were as follows: (1) there is no difference in translucency and colour between Filtek Z250 XT (Nano Hybrid composite), Cention N (alkasite based composite resin) and SDR Plus Bulk Fill Flowable (low viscosity bulk fill material), and (2) there is no difference in staining potential between different beverages (distilled water, Coca-Cola, chocolate milk, and orange juice) with time on different restorative materials.

## Methods

The present research was conducted in the Faculty of Dentistry, Alexandria University following CRIS guidelines for reporting in-vitro studies (Krithikadatta et al. 2014). Ethical approval was obtained from the Institutional Ethical Committee, Alexandria University, Egypt. (IRB # 0852-1/2024-IORG 0008839).

### Sample size

The sample size for the study was calculated using a power analysis based on several factors, including a 5% significance level (alpha error), 80% study power (1—beta error), and the variance explained by the effects of composite material, immersion liquid, and immersion time on colour differences, as reported by Karakaş and Küden (2023). The specific variance explained by these factors was estimated to be 0.865. To calculate the sample size, a two-way repeated measures analysis of variance (ANOVA) was used. Using G*Power 3.1.9.7, the sample size was determined to be 12 samples per group. Hence, the total number of samples = sample per subgroup (12) x Number of subgroups (4) x number of groups (3) = 144.

### Sample preparation

Forty-eight discs (10-mm diameter × 2-mm thickness) of shade A2 were prepared from each of the three restorative material under investigation in this study giving a total of 144 samples (Tan et al. 2015). These specimens were classified into Group I: Filtek Z250 XT (Nanohybrid, 3 M ESPE, USA), Group II: Cention N (Alkasite composite resin, Ivoclar Vivadent, Schaan, Liechtenstein), and Group III: SDR flow Plus Bulk Fill Flowable (Low viscosity bulk fill material, Dentsply, Germany). (Table 1).

**Table 1:** Resin composite material used in the study

Material	Type	Composition	Filler content	Manufacturer
Filtek ™ Z250 XT	Nanohybrid	BISGMA, UDMA, BISEMA, TEGDMA. Zirconia/Silica particle	*82% by weight. (68% by volume)*	3 M ESPE,USA
Cention N	Alkasite Dual cure bulk fill	UDMA Calcium fluorosilicate glass, calcium barium aluminum Ytterbium trifluoride	78% by weight (58% by volume)	Ivoclar, Liechtenstein
SDR® Plus Flowable	Low viscosity bulk fill	Modified UDMA, TEGDMA, dimethacrylate, trimethacrylate resins	47% by volume	Dentsply, Germany

*BisGMA* Bisphenylglycidyl dimethacrylate;

*UDMA* Urethane dimethacrylate

*BisEMA* Ethoxylated bisphenol-A dimethacrylate

*TEGDMA* Triethylene glycol dimethacrylate.

The discs were fabricated using a specially designed metal mould. It consisted of a plastic base, two split metal plates and a plastic cover. A transparent mylar strip (Kerr, USA) was placed between the plastic base and the metal plates, below the mould (Tan et al. 2015; Karadas 2016). According to the manufacturer, the transparent strip is 10-cm long, 1-cm wide and 0.05-mm thick. According to the manufacturers’ instructions, the materials were manipulated and packed into the mould, another mylar strip was placed above the metal plate (Karadas 2016; Gonder and Fidan 2022). A 1 mm thick glass slide was placed over the strip and slight finger pressure was applied to obtain a smooth surface and to remove excess material to flatten the surfaces and care was taken to prevent entrapment of air voids within the specimens. (Karakaş and Küden 2023; Karadas 2016; Gonder and Fidan 2022; Al-Haj et al. 2021).

### Polymerization

Upon polymerization, the tip of the light unit was placed on the glass slide to ensure that curing was performed from a standardized distance (Elwardani et al. 2019; Bezgin et al. 2015). Polymerization was performed using LED curing unit (Blue phase G2, Ivoclar Vivadent, USA) in standard mode with an intensity of 1200 mW/cm^2^. The samples were cured through the Mylar strip for 20 s to each surface for a total of 40 s according to the manufacturers’ instructions (Al-Haj et al. 2021; Bezgin et al. 2015). Every five samples the output of the light was checked using a photometric tester (Bluephase Meter II, Ivoclar Vivadent, USA) (Bahbishi et al. 2020). Then, the samples were removed from the mould and were visually inspected for surface defects, those with visible flaws were discarded. The included discs were stored in 20 ml of saliva natura (Medac, UK) at 37 °C for 24 h in an incubator to ensure stabilisation of monomer conversion and to mimic oral conditions (Habib et al. 2017). Then the composite discs were washed thoroughly by distilled water for 10 s and left to dry.

### Allocation and blinding

The prepared samples from each main group were placed in identical numbered containers by an independent colleague (NS), who randomly assigned the specimens into subgroups according to the immersion solution used. Allocation was performed by a computer-generated list of random numbers to create block randomization. Hence, each main group was subdivided into 4 subgroups (*n* = 12), classified as; Subgroup A (control): distilled water (Runyes water distiller, China), Subgroup B: Coca-Cola (Coca-Cola, Egypt), Subgroup C: chocolate milk (Juhayna, Egypt), and Subgroup D: Orange juice (Juhayna, Egypt). Double blind study was implemented since allocation was concealed from both the investigator and statistician (Elwardani et al. 2019).

After baseline measurements, each sample was immersed in undiluted 20 ml of the assigned beverage for 10 min every day. Next samples were washed thoroughly for 10 seco by distilled water. Then samples were re-immersed in saliva natura, which acts as a storage medium, and placed back in the incubator at 37 °C. Every day the immersion solutions were renewed (Mundim et al. 2010).

### Optical evaluation

Assessment was done before immersion (baseline) and on day 30 of exposure (Al-Haj et al. 2021). Translucency and colour measurements were performed with a digital spectrophotometer (VITA Easyshade Advance, Vita Zahnfabrik, Germany) according to CIE-Lab system (Commission International de L’Eclairage), that is recommended by the American Dental Association (Karadas 2016; Habib et al. 2017). In which, any colour is expressed through L^*^, a^*^, and b^*^ values. The L^*^ represents the measurement along the white-black axis (0: black, 100: white), a^*^ refers to the measurement along the red-green axis (− a^*^: green, + a^*^: red) and b^*^ denotes the measurement in the yellow-blue axis (− b^*^:blue, + b^*^:yellow). Colourimetric measurements through the CIE-Lab coordinates allows the determination of colour in three-dimensional space (Arregui et al. 2016).

Before measuring, the Easyshade Vita colourimeter was calibrated according to the manufacturer’s instructions. The probe tip was placed perpendicular and well-adjusted to the specimens’ surface to make accurate measurements. Three successive readings were taken on each surface for every specimen, and the average value was recorded. Calibration was repeated every 4 specimens (Karakaş and Küden 2023; Salgado et al. 2018; Hatirli et al. 2022).

### Translucency assessment

Translucency Parameter (TP) was determined by calculating the difference between colour coordinates values obtained for the same specimen against black (L^*^_B_ = 4.2, a_B_^*^ = 0.3, b_B_^*^ = -1.2) and white backgrounds (L^*^_W_ = 93.3, a^*^_W_ = − 0.1, b^*^_W_ = 2.6) according to the following formula: (Karadas 2016; Salgado et al. 2018).

$$ {\text{TP}}\, = \, \, \left[ {\left( {{\text{L}}^{*}_{{\text{B}}} {-}{\text{L}}^{*}_{{\text{W}}} } \right) + \left( {{\text{a}}^{*}_{{\text{B}}} {-}{\text{a}}^{*}_{{\text{W}}} } \right)^{2} + \left( {{\text{b}}^{*}_{{\text{B}}} {-}{\text{b}}^{*}_{{\text{W}}} } \right)^{2} } \right]\,^{{{1 \mathord{\left/ {\vphantom {1 2}} \right. \kern-0pt} 2}}} $$

Where the subscript _W_, refers to the colour coordinate values obtained against a white background and the subscript _B_, refers to the values against the black background. The difference in TP (∆TP) was calculated by the equation: (Tan et al. 2015).

$$ \Delta {\text{TP}}\, = \,{\text{TP}}_{{\text{A}}} {-}{\text{TP}}_{{\text{B}}} $$

In this equation, subscript _A_, refers to baseline measurements and subscript _B_, refers to measurements after immersion in the beverage (Karadas 2016; Kang et al. 2015).

### Colour change assessment:

Colour measurements were obtained over the white background to prevent potential absorption effects on any of colour parameters (Karadas 2016; Habib et al. 2017). The colour difference (∆E) was calculated as follows: (Karadas 2016).

$$ \Delta {\text{E}}\, = \left[ {\left( {\Delta {\text{ L}}} \right)^{2} + \left( {\Delta {\text{a}}} \right)^{2} + \left( {\Delta {\text{b}}} \right)^{2} } \right]\,^{{{1 \mathord{\left/ {\vphantom {1 2}} \right. \kern-0pt} 2}}} $$

Where ∆L, ∆a, and ∆b are differences in L^*^, a^*^ and b* values between the baseline and after 30 days of exposure (Al-Haj et al. 2021).

## Results

### Statistical analysis

Normality was checked using Shapiro wilk test and Q-Q plot. TP was normally distributed whist ∆E was not normally distributed. Thus, mean and standard deviation were used to present TP whist ∆E was presented using median and inter quartile range (IQR). Two Way Repeated Measures ANOVA was performed to assess the main effect and interaction of material type and immersion medium on TP at different immersion times, this was followed by pairwise comparisons with Bonferroni correction. Kruskal–Wallis test followed by post hoc test with Bonferroni correction was performed to compare differences in ∆E between immersion medium for each material and between materials immersed at different mediums. All tests were two tailed and the significance level was set at p value ≤ 0.05. Data were analyzed using IBM SPSS version 23, Armonk, NY, USA.

**Fig. 1:**
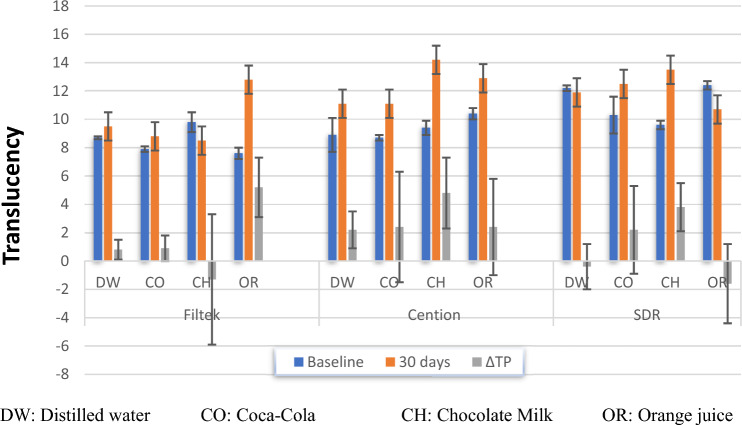
Mean and standard deviation of TP values

The mean TP values and standard deviations are presented in Fig. 1. At baseline, SDR was the most translucent in comparison to Cention N and Filtek Z250 XT.

Table 2 shows the effect of different factors and their interaction on TP. There was a statistically significant influence of restorative material, immersion time and immersion solution on the change of translucency either independently (*p* < 0.0001*, *p* < 0.0001*, *p* = 0.001,* respectively) or as combined factors (*p* = 0.001*, *p* = 0.022*, *p* < 0.0001*). However, the effect of different immersion solution with time on the change of translucency showed no statistical difference within different restorative materials under investigation (*p* = 0.089).

**Table 2:** Effect of different factors and their interaction on TP

Variables	df	Mean squares	F test	*p* value	Ƞ^2^
Immersion time	1	230.3	65.3	< 0.0001*	0.33
Restorative material	2	146.1	40.2	< 0.0001*	0.38
Immersion solution	3	21.2	5.8	0.001*	0.12
Immersion time x restorative material	2	25.5	7.2	0.001*	0.10
Immersion time x immersion solution	3	7.8	2.2	0.089	0.05
Restorative material x immersion solution	6	9.4	2.6	0.022*	0.11
Immersion time x restorative material x immersion solution	6	41.2	11.7	< 0.0001*	0.35

*Statistically significant difference at *p* value ≤ 0.05 Ƞ^2^: Partial Eta Squared

The pairwise comparison between groups was shown in Table 3. Regarding immersion time, there was a significant change in translucency between baseline and after 30 days of immersion in different solutions (*p* < 0.0001).

**Table 3:** Pairwise comparison between groups

Variables	Groups	Compared to	*p* value
Immersion time	Baseline	30 days	< 0.0001*
Restorative material	Filtek Z250 XT	Cention N	< 0.0001*
		SDR	< 0.0001*
	Cention N	SDR	0.013*
Immersion solution	Distilled water	Cola	0.589
		Chocolate milk	1.00
		Orange juice	0.155
	Cola	Chocolate milk	0.018*
		Orange juice	0.001*
	Chocolate milk	Orange juice	1.00

*Statistically significant difference at *p* value ≤ 0.05

Also, there was a statistically significant differences in translucency between the 3 restorative materials (*p* < 0.0001*, *p* < 0.0001*, *p* = 0.013*). At baseline, SDR showed significantly higher translucency readings, followed by Cention N and Filtek Z250 XT respectively. However, after 30 days of immersion, Cention N had the significant higher translucency readings than SDR followed by Filtek Z250 XT in that order (Fig. 1).

However, regarding the different immersion solution, there was only statistically significant effect on the change of translucency of the restorative materials between Coca-Cola and chocolate milk (*p* = 0.018*), likewise between Coca-Cola and orange juice (*p* = 0.001*).

**Fig. 2:**
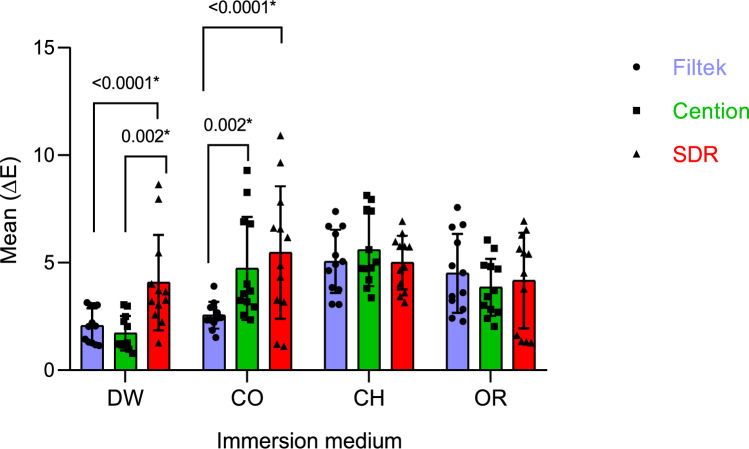
Comparisons of ∆E between restorative materials for each immersion solution

**Fig. 3:**
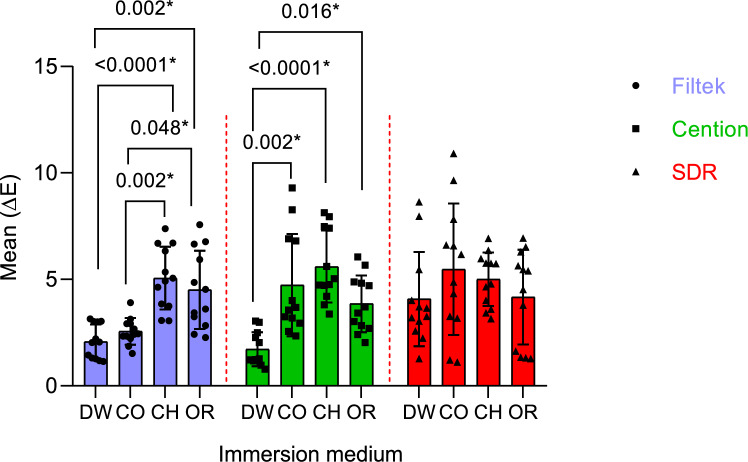
Comparisons of ∆E induced by different immersion solutions for each restorative material

As for colour change, the descriptive values of (∆E) fare shown in Table 4, whist pairwise comparison is represented in Figs. 2 and 3.

**Table 4:** Descriptive statistics of ∆E for the restorative materials

Restorative material	Immersion solution								*p* value
	Distilled water		Cola		Chocolate milk		Orange juice		
	Mean ± SD	Median (IQR)	Mean ± SD	Median (IQR)	Mean ± SD	Median (IQR)	Mean ± SD	Median (IQR)	
Filtek Z250 XT	2.1 ± 0.8	2.1 (1.7)	2.6 ± 0.6	2.4 (0.7)	5.1 ± 1.5	5.0 (2.8)	4.5 ± 1.8	4.1 (3.5)	< 0.0001*
Cention N	1.7 ± 0.8	1.3 (1.3)	4.7 ± 2.4	3.6 (3.9)	5.6 ± 1.7	4.9 (3.1)	3.9 ± 1.3	3.6 (2.1)	< 0.0001*
SDR	4.1 ± 2.2	3.5 (2.4)	5.5 ± 3.1	5.5 (4.3)	5.0 ± 1.3	5.3 (2.2)	4.2 ± 2.2	5.0 (4.7)	0.426
***P* value**	< 0.0001*		0.003*		0.702		0.695		

*Statistically significant difference at *p* value ≤ 0.05

Table 4 shows a statistically significant effect of different immersion solution (distilled water, Coca-Cola, chocolate milk and orange juice) on colour change within Filtek Z250 XT and Cention N (*p* < 0.0001, p < 0.0001), but no statistically significant effect on colour change for SDR (*p* = 0.426). Moreover, distilled water and Coca-Cola had a significant effect on colour change for all restorative material under investigation (Filtek Z250 XT, Cention N, and SDR). Whist chocolate milk and orange juice showed no statistically significant effect on colour changes among the different restorative materials.

The statistical comparison of the change in colour between different restorative materials under investigation when immersed in the same solution is seen in Fig. 2. When immersed in distilled water, SDR showed a significantly higher colour change when compared to Filtek Z250 XT (*p* < 0.0001*), as well as Cention N (*p* = 0.002). Whist Cola resulted in a significantly higher colour change for SDR (*p* < 0.0001) and Cention N (*p* = 0.002) when compared to Filtek Z250 XT. On the other hand, neither chocolate milk nor orange juice caused significant change in colour for all restorative materials.

The statistical comparison of the change in colour between different immersion solutions on each restorative material is expressed in Fig. 3. Filtek Z250 XT showed a significantly higher colour change in chocolate milk, as well as orange juice when compared to Coca-Cola (*p* = 0.002, *p* = 0.048) and distilled water (*p* = 0.001, *p* = 0.002). Whist Cention N had a significant higher colour change in chocolate milk, Coca-Cola and orange juice when compared to distilled water (*p* < 0.001, *p* = 0.002, *p* = 0.016). In Contrast to SDR which showed no statistically different colour change between all immersion solution under investigation.

## Discussion

Resin-based restorative are known for having colour and optical instability during clinical service (Karakaş and Küden 2023). The current study implemented the use of shade A2 as it is the only shade available for Cention N, it is often used in similar studies (Karadas 2016). Despite the fact that, shade A1 is the most selected shade for primary teeth (Kim et al. 2007; Tirapelli 2022). For standardization, a polyester mylar strip was used to achieve the smoothest surface possible which is representative of the clinical situation when matrices are utilized. Moreover, Mylar finish eliminates the need for polishing and minimized operator variability (Tan et al. 2015). To ensure the accuracy of colour measurements, in this study, the specimens’ diameter was equal to the spectrophotometer’s observation area, hence; the edge-loss effect is negligible (Salgado et al. 2018). Moreover, the present study used distilled water, although saliva would be expected to present a much better protective effect (Arregui et al. 2016). Natural saliva forms a surface barrier that limits staining and dilutes staining solutions (Omata et al. 2006; Bagheri et al. 2005). However, there is no way to simulate the fresh saliva.

In the current study, SDR had the highest translucency at baseline, this can be related to reduced filler content and increased filler size. Thus the surface between fillers and organic matrix is lowered, so reducing light scattering (Bucuta and Ilie 2014).

Cention N has a high filler content with the incorporation of highly reactive fillers especially in acidic environment; isofillers (Alla et al. 2023). These are fillers embedded in a prepolymerized organic matrix, resulting in approximating the refractive index of fillers to that of the resin matrix (Gonder and Fidan 2022). This leads to less scattering of light, allowing greater light penetration into the material and increasing translucency (Alla et al. 2023). Cention N releases hydroxide ions to regulate pH preventing demineralization, in addition to the release of fluoride and calcium enhancing remineralization (Alla et al. 2023). Hence, Cention N displays high translucency values at baseline as well as after immersion in different solutions.

Interestingly, there was a significant increase in overall translucency in all material after immersion, except for Filtek Z250 XT immersed in chocolate milk, as well as SDR immersed in distilled water and orange juice. The hydrolytic degradation of composite restorations overtime created free spaces filled with water, colourants of chocolate milk and orange juice leads to a mismatch between the refractive index of the polymer than that of water consequently lowering translucency (Piccoli et al. 2019). The result of the current study is in disagreement with previous findings which resulted in a decrease in translucency. This can be related to the limited sample size and short duration implement by Kang et al. (2012) and Karadas (2016).

The authors of this study followed ∆TP_ab_ formula that was reported by Paolone et al. (2022), as it is considered to be the most frequently used formula (> 90%). Salas et al. (2018), marked the detectability and acceptability thresholds of ∆TP_ab_ as 1.3 and 4.4, respectively. Considering the results of the current study, only Filtek Z250 XT immersed in orange juice and Cention N immersed in chocolate milk demonstrated clinically unacceptable change in translucency.

As for colour change, the acceptability and perceptibility thresholds vary and are corelated to their clinical significance (Paolone et al. 2022; Alshali et al. 2015). There are three intervals to distinguish ∆E values; value of ∆E < 1 is imperceptible to the human eye, value between 1 < ∆E < 3.3 is perceptible by skilled operators, but still clinically acceptable. Whist value of ∆E > 3.3 is not clinically acceptable (Elwardani et al. 2019). The colour changes for all composite materials in this study were considered clinically unacceptable (∆E > 3.3). Except for the Filtek Z250 XT immersed in distilled water and cola were clinically acceptable as well as Cention N immersed in distilled water (∆E < 3.3). Whist the greatest colour change was detected within SDR composite and chocolate milk.

In accordance with Habib et al. (2017), there was no significant difference in colour change between different restorative materials immersed in chocolate milk and orange juice. The greasy nature and dark pigments of chocolate milk causes surface adsorption of colourants. Whist the low pH of orange drinks (pH = 3.5) leads to more surface roughness enhancing accumulation of residues and dyes (Bezgin et al. 2015; Habib et al. 2017).

Similar to the results of Karakaş and Küden (2023), Coca-Cola presented similar colour change for SDR and Cention N, significantly higher than Filtek Z250 XT. The larger filler size content of Cention N and the presence of selfcure initiators inducing coloured oxidation product cause intrinsic discolouration (Karakaş and Küden 2023). However, the result disagrees with Mundim et al. (2010), in which there was no significant difference in colour change between Filtek Z250 and SDR after 15 days of continuous immersion. Such varying results can be attributed to the reduced sample size and different pattern of immersion implemented by Mundim et al. (2010).

Whereas, distilled water exhibited significant higher change in colour for SDR than Filtek Z250 XT and Cention N. The least colour change in Cention N could be related to the absence of TEGDMA in its constituents, unlike SDR and Filtek Z250 XT (Alla et al. 2023). Previous studies accused TEGDMA of increasing water sorption, in addition to its heterogenic structure forming micro-pores between the polymer which is consequently occupied by water (Alshali et al. 2015).

There is a disagreement with Mundim et al. (2010) results, which concluded that there was no significant difference in colour change between SDR and Filtek Z250 after continuous immersion in distilled water for 15 days. This can be attributed to their reduced sample size, surface polishing and different pattern of immersion Yet, these results agree with Hatirli et al. (2022), which concluded no significant colour change between Filtek Z250 and Cention N after immersion in distilled water for 28 days.

Regarding the effect of different beverages on each material individually, SDR—in accordance with Mundim et al. (2010), showed no significant change in colour between all immersion solutions under investigation. Whereas for Cention N, only immersion in distilled water showed a significantly lower colour change when compared to other immersion solution. To the best of the authors knowledge, there are no studies evaluating the effect of similar beverages on colour change of Cention N. As for Filtek Z250 XT, the results of the present study is in compliance with previous studies which concluded that immersion in chocolate milk and orange juice induced a significantly higher colour change than cola drink and distilled water (Al-Haj et al. 2021; Al-Anesi et al. 2019). Frequent intake of oral fluids greatly affects the optical match of restorative material, hence jeopardises aesthetics.

The limitations of the study included the inability of the in-vitro model to completely replicate the complex oral environment. Restorative materials in the mouth are constantly exposed to colourants from food and beverages, which are then dispersed in saliva. For future research, it is important to consider other factors that can contribute to colour stability, such as proper polymerization, surface protection, finishing, and polishing techniques. Additionally, the extent of discolouration caused by beverages on restorative dental materials are influenced by the pH values and sugar content of the beverages. According to the current research, resin-based restorative materials had the ability to absorb water and other beverages having colourants, which cause alteration in optical properties of the material. Overall, these findings emphasize the importance of considering the type of restorative material, putting in consideration the frequent consumption of coloured beverages can help in selecting appropriate materials and optimizing clinical outcomes.

## Conclusion

Considering the limitation of the present study, it has been concluded that the translucency and colour stability of resin based restorative materials were notably affected by frequent consumption of coloured beverages. The optical properties of restorative materials are significantly influenced by the type of restorative material, immersion time, and immersion solution.

## Data Availability

All data generated or analysed during this study are included in this published article and its supplementary information file.
